# Advanced optical waveguide design *via* encapsulation of 2,4,6-triphenylpyrylium chloride in oxide glasses

**DOI:** 10.1039/d5nr02213d

**Published:** 2025-07-31

**Authors:** Eleni Agapaki, Ioannis Konidakis, Egor Evlyukhin, Klytaimnistra Katsara, Georgios Kenanakis, David King, Haesook Han, Pradip K. Bhowmik, Emmanuel Stratakis

**Affiliations:** a Institute of Electronic Structure and Laser (IESL), Foundation for Research and Technology-Hellas (FORTH) 70013 Heraklion-Crete Greece ikonid@iesl.forth.gr stratak@iesl.forth.gr; b Department of Chemistry and Biochemistry, University of Nevada Las Vegas Las Vegas Nevada 89154 USA

## Abstract

Pyrylium ion (C_5_H_5_O^+^)-based salts exhibit distinctive optical properties that can be tuned by external stimuli such as temperature and pressure, making them suitable materials for various nanoscale optoelectronic applications. However, their practical use has been limited by their solid powder form, which poses challenges for integration into realistic devices. Herein, we present a low-temperature, post-melting encapsulation method for the incorporation of a 2,4,6-triphenylpyrylium chloride salt within transparent phosphate glasses containing dispersed silver nanoparticles. This synthesis approach enables spatially controlled vitrification of high-refractive index pyrylium pathways within the glass matrix. The encapsulated salt retains its structural and optical properties, while the presence of randomly dispersed silver nanoparticles enhances light transmission upon scattering effects. The resulting pyrylium salt-glass composites exhibit robust waveguiding characteristics, positioning this technique as a promising route for the fabrication of advanced nano-engineered optoelectronic devices.

## Abstract 2

Post-glass melting encapsulation of pyrylium ion based salts in inorganic oxide glasses for the development of advanced optical waveguides.

## Graphical abstract

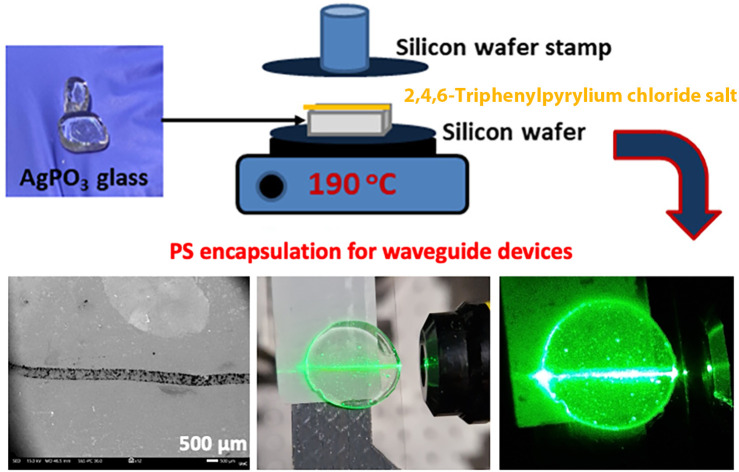

## 1. Introduction

Pyrylium salts are six-membered cationic heterocyclic compounds featuring a positively charged oxygen and a variety of counterions, such as Cl^−^, ClO^4−^, BF^4−^, PF^6−^, TsO^−^, Tf_2_N^−^, and FeCl^4−^.^1^ These salts are known for their remarkable absorption, fluorescence, and electron transfer capabilities, making them highly useful in various applications, such as light emitters,^2^ photocatalysts,^3^ photosensitizers in organic transformations, synthesis of heterocyclic compounds, complex macrocycles, and metallo-supramolecular structures.^4–7^ Additionally, they have been used to facilitate radical and cationic photopolymerization reactions and have been employed in metal-free ring-opening metathesis polymerization processes.^6–9^ Most recently, the piezochromic behavior of pyrylium salts was demonstrated using a diamond anvil cell,^10^ a device that enables the study of materials under extreme pressures.^11,12^ Despite their significant potential, a key limitation lies in their typical solid powder form, which necessitates dissolution in suitable solvents for effective processing. This immediately hinders their potential towards photonic and optoelectronic devices. In addition, pyrylium salts’ solubility can be restrictive in some non-polar solvents, which also hinders their further use in specific chemical systems or reactions.^2^

To overcome these limitations, we herein demonstrate a fast, low-temperature, post-melting encapsulation (PME) fabrication procedure that allows the incorporation of pyrylium salts in transparent inorganic oxide silver phosphate glasses. The developed PME approach offers significant benefits, including the versatility of utilizing pyrylium-based materials and their useful optical properties in both soluble and powder forms. Notably, the PME has been employed successfully in other cases yielding highly effective results. For instance, the encapsulation of cesium lead bromide perovskite nanocrystals resulted in their exceptional photoluminescence (PL) stability when compared to identical perovskite crystals exposed to ambient air.^13^ Similarly, silver phosphate glass-embedded two-dimensional (2D) molybdenum disulfide (MoS_2_) crystals have shown tailored PL properties upon interaction with the glass network and the randomly present silver nanoparticles (NPs).^14^ Notably, the host silver phosphate (AgPO_3_) glass is known for its exceptionally low glass transition temperature of 192 °C, reflecting its soft and malleable nature. Meanwhile, in this study, the employed 2,4,6 triphenylpyrylium chloride salt begins to degrade at temperatures above 225 °C,^15^ thereby making the AgPO_3_ glass an excellent host matrix for encapsulating these compounds.

The successful spatially controlled encapsulation of pyrylium salts in the phosphate glass allows the feasible formation of optical waveguides suitable for photonic circuits. Currently, the fabrication of glass optical waveguides relies mainly on two technologically demanding approaches: one involves modifying the refractive index of glass employing laser processing and chemical etching techniques,^16^ while the other depends on precise and complex thin-film deposition processes.^16^ This work significantly simplifies the development of glass-based optical waveguides as pyrylium salts are feasibly utilized to spatially modify the refractive index of glass. By this means, high refractive index light transmission pathways are formed across the pyrylium composite glasses in a controlled manner. To the best of our knowledge, no prior studies have demonstrated the direct integration of pyrylium salts into inorganic glass waveguides. This research thus fills a critical gap, introducing a practical pathway to effectively utilize the optical functionalities of pyrylium salts in stable, long-term optoelectronic and photonic devices.

## 2. Experimental

### 2.1 Synthesis of silver phosphate glass and the 2,4,6-triphenylpyrylium chloride salt

For the synthesis of silver metaphosphate glass (AgPO_3_), high purity AgNO_3_ (99.995%) and NH_4_H_2_PO_4_ (99.999%) powders were employed in a platinum crucible, following a well-established synthesis procedure.^13,17^ The resulting AgPO_3_ glass was shaped into 3 mm thick disk specimens with a diameter of about 1 cm by rapidly quenching the melt between two silicon wafers. This process produced smooth glass surfaces, making the samples ideal substrates for encapsulating the pyrylium chloride salt. The 2,4,6-triphenylpyrylium chloride salt (PS) was also synthesized following a previously reported procedure.^18^

### 2.2 Post melting encapsulation (PME) technique

**Fig. 1:**
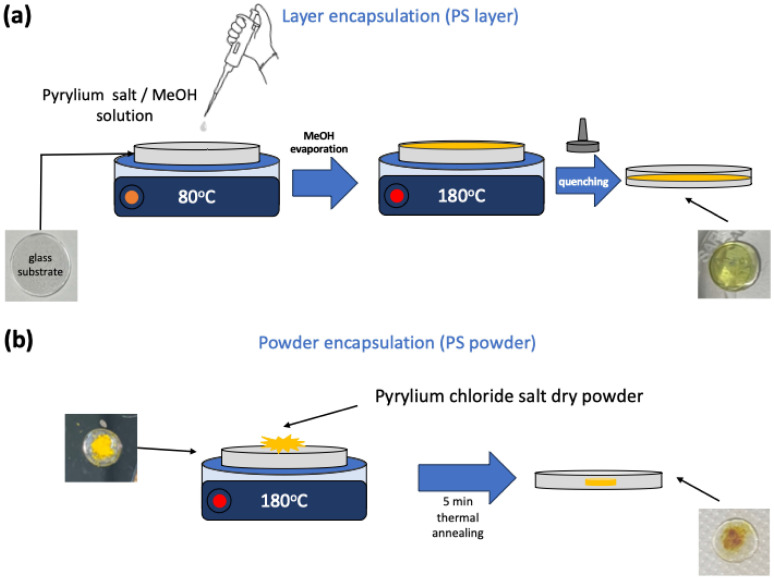
Schematic illustration of the PS layer (a), and the PS powder (b) encapsulation in silver phosphate glass.

The PME technique was used to embed the 2,4,6-triphenylpyrylium chloride salt (PS) into a glass matrix, resulting in two types of composite AgPO_3_ glasses based on the initial salt form, *i.e.*, the PS layer and PS powder. Fig. 1 provides a schematic illustration of the encapsulation methods for both types of samples. For the PS layer, appropriate amounts of PS were first diluted in methanol (MeOH) for the formation of solutions with various concentrations ranging from 1 × 10^−3^ M to 1 × 10^−1^ M (Fig. S1). Fig. S1 depicts the PS layer formation on typical microscope slides upon drop casting 50 μL of the solutions following MeOH evaporation. Fig. S2 presents the Raman spectra of the drop-cast solid PS residues from PS/MeOH solutions after the evaporation step. As seen in Fig. S2, the PS retains its molecular characteristics regardless of the concentration of the MeOH solution used (Fig. S1). Accordingly, the PS layer composite glasses were prepared upon drop-casting 50 μL of 1 × 10^−3^ and 1 × 10^−2^ M solutions on the phosphate glass surface kept at 80 °C (Fig. 1a). 50 μL of PS/MeOH solution were added in the form of droplets with 10 s intervals for gentle solvent evaporation. Afterwards, the temperature was raised to 180 °C, *i.e.*, near the glass transition temperature (*T*_g_ = 192 °C) of the AgPO_3_ glass.^19^ At this temperature, the glass substrate gains viscosity and allows the PS layer to be incorporated beneath the glass surface upon splat-quenching with a silicon wafer (Fig. 1a). Fig. S3a and b depict indicative photos of the composite PS layer glasses upon using 1 × 10^−3^ and 1 × 10^−2^ M PS/MeOH solutions, respectively.

Similarly, the PS powder samples were synthesized by placing approximately 3 mg of dry PS powder directly on the surface of the phosphate glass. After heating the glass at 180 °C for a few seconds, the composite PS powder glass was splat-quenched within two silicon wafers to incorporate the PS powder inside the glass. The PS powder composite glass was then removed from the hot plate allowing it to cool down to room temperature. Typical PS powder glasses are depicted in Fig. 1b and Fig. S3c. It is noted that, following PS encapsulation, no further annealing is performed on the composite PS-glass devices.

### 2.3 Characterisation techniques

The obtained composite PS layer and PS powder composite glass samples were characterised by using scanning electron microscopes (SEM, JEOL JSM-7000F and JSM-6490) paired with an INCA PentaFET-x3 EDS detector. To investigate changes in the phosphate glass network resulting from the incorporation of PS encapsulation, room temperature Raman spectroscopy was conducted using a LabRAM HR Evolution confocal Raman microscope (LabRAM HR; HORIBA FRANCE SAS, Lille, France). A 785 nm laser line was used for Raman excitation, with the laser output power set to approximately 400 mW. The instrument's spectral resolution, achieved through 600 grooves per mm grating, was around 2 cm^−1^. An Olympus 50× objective lens (LMPlanFLN 50×/0.5, Olympus) was utilized. For absorption studies, a Shimadzu UV-1700 spectrometer was employed, utilizing quartz cuvettes with a 0.2 cm path length and covering an overall wavelength range of 200–800 nm.

## 3. Results and discussion

The selected photosensitizer, 2,4,6-triphenylpyrylium chloride, possesses a high melting point, reported in the literature as being between 217 °C and 225 °C.^20^ This thermal stability makes it an excellent candidate for encapsulation processes, where maintaining structural integrity at elevated temperatures is crucial. Firstly, the PS powder residue from the drop-cast PS/MeOH solution was placed on a microscope slide and heated at 180 °C for several minutes. As shown in Fig. S3d, optical inspection of the sample reveals no significant color changes or visible melting. Secondly, the PS was dissolved in MeOH and drop-cast on the phosphate glass (Fig. S4a and b) for solvent evaporation under two conditions: at room temperature (Fig. S4c and d) and on a hot plate at 80 °C (Fig. S4e and f). Inspection of SEM photos reveals no significant differences between the two samples, indicating that the PS can easily sustain the solvent evaporation temperature of 80 °C following dissolution in MeOH without obvious changes in the structural morphology and crystal orientation (Fig. S4).

**Fig. 2:**
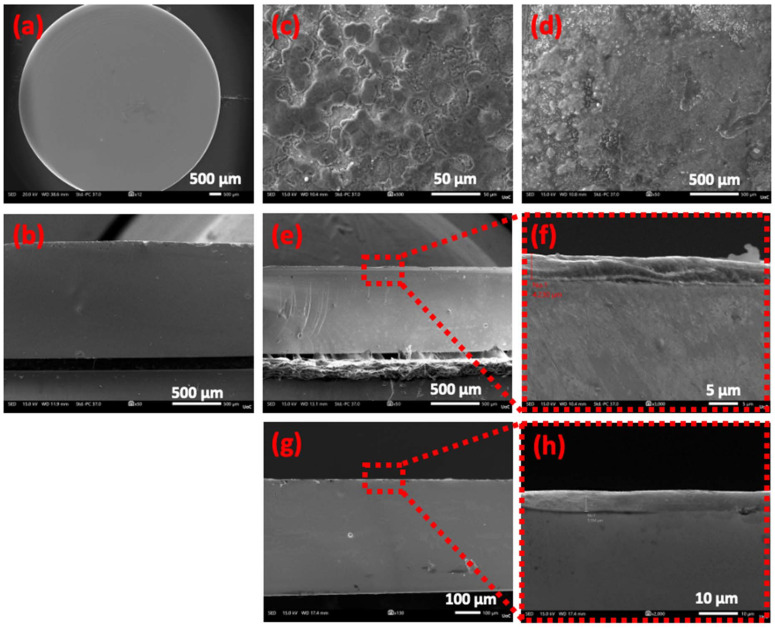
SEM images showing: (a) a top view of the AgPO_3_ pristine glass. (b) A cross section of the AgPO_3_ pristine glass. (c) A top view of the PS layer-glass (*C* = 10^−2^ M). (d) A top view of the PS powder glass. (e) A cross section of the PS layer-glass (*C* = 10^−2^ M), along with a magnified image of the same cross-section (f). (g) A cross section of the PS powder-glass, along with a magnified image of the same cross-section (h).

Fig. 2a depicts an SEM image of the surface, whereas Fig. 2b shows the corresponding cross-sectional image of the AgPO_3_ glass. Both photos reveal the outstanding uniformity of the phosphate glass substrate. Similarly, the top views of the so-formed composite PS layer and PS powder glasses are presented in Fig. 2c and d, respectively. Namely, the top view SEM photos reveal an intense presence of the PS salt vitrified on the surface of both types of composite glasses (Fig. 2c and d), when compared to the smooth surface of the pristine phosphate glass (Fig. 2a). Fig. 2e and f present images of the cross-sectional views of the PS layer sample. Notably, the PS layer is integrated into the glass matrix within a depth of ∼5 μm (Fig. 2f), an observation supported by electron diffraction spectroscopy (EDS) (Fig. S5). Such thickness is the minimum that could be obtained for achieving a homogeneous incorporation of a uniform PS layer inside the glass. Indeed, the EDS data obtained from the cross-sectional area reveal a clear elimination of Cl atoms, originating from the PS, as the probing point transitions from the vitrified PS layer in glass (Fig. S5a) to the pristine bulk phosphate glass (Fig. S5g). This observation is consistent with SEM images revealing a PS encapsulation depth of ∼5 μm (Fig. 2f), leaving only the elemental components of the AgPO_3_ glass detectable beyond this depth. Finally, Fig. 2g and h present the corresponding cross-sectional surfaces of the PS powder-glass. The SEM photos indicate that the PS powder was successfully vitrified within the glass matrix, forming an embedded PS core with a maximum depth of around 10 μm (Fig. 2h).

**Fig. 3:**
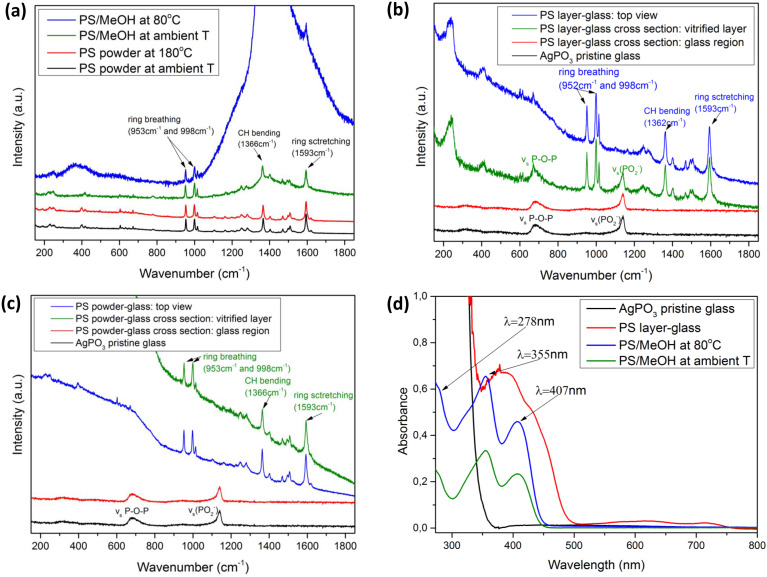
(a) Raman spectra of the 2,4,6-triphenylpyrylium chloride powder salt both at ambient temperature, temperature treated, 2,4,6-triphenylpyrylium chloride salt diluted in MeOH (*C* = 10^−2^ M) at ambient temperature, and heated at 80 °C. (b) Raman spectra of the AgPO_3_ pristine glass, cross-sectional glass region of the PS layer-glass, the cross-sectional vitrified layer of the PS layer-glass, and the top view of the PS layer-glass. (c) Raman spectra of the AgPO_3_ pristine glass (black line), the glass region of the PS powder-glass (red line), the cross-sectional vitrified layer of the PS powder-glass, and the top view of the PS powder-glass. (d) Optical absorbance of the AgPO_3_ pristine glass, 2,4,6-triphenylpyrylium chloride salt (*C* = 10^−2^ M) encapsulated in the AgPO_3_ glass, 2,4,6-triphenylpyrylium chloride salt diluted in MeOH (*C* = 10^−5^ M), and 2,4,6-triphenylpyrylium chloride salt diluted in MeOH and heated at 80 °C.

Raman spectroscopy was utilized to investigate potential structural changes in the phosphate glass network and PS caused by the encapsulation of the latter inside the glass matrix. Fig. 3a presents the Raman spectra of the PS powder at room temperature and after heating to the encapsulation temperature of 180 °C. The spectra appear identical while exhibiting the characteristic key peaks at 953 cm^−1^, 998 cm^−1^, 1366 cm^−1^, and 1593 cm^−1^, attributed respectively to the aromatic ring breathing, C–H in plane bending, and benzene ring stretching.^10,21^ Moreover, when the PS was dissolved in MeOH, following the slow solvent evaporation at room temperature and 80 °C, the position of the PS characteristic peaks remained unchanged. However, the heated sample exhibits an intense fluorescence profile in the 1200–1800 cm^−1^ range, overlapping the CH bending peak.^22^ Nevertheless, the clear presence of the ring breathing and stretching peaks implies the molecular and structural unity of the PS salt residue upon heating the PS/MeOH solution for solvent removal. These observations verify that the employed PS salt can sustain the PME annealing conditions for the development of both the PS layer and PS powder composite glasses.

Indeed, Fig. 3b depicts room-temperature Raman spectra of the pristine AgPO_3_ glass and the so-formed composite PS layer glass. The metaphosphate network of the glass is primarily composed of chains formed by interconnected phosphate tetrahedral units, featuring both bridging and non-bridging (terminal) oxygen atoms. Specifically, the prominent band at approximately 1144 cm^−1^ is attributed to the symmetric stretching vibration of terminal PO_2_ groups (v_s_(PO_2_^−^)), while the broader band near 675 cm^−1^ is associated with the symmetric stretching of P–O–P bridges within the phosphate backbone (v_s_(P–O–P)).^17^ Notably, the cross-sectional Raman spectrum of the composite PS layer glass beneath the PS-encapsulated 4 μm layer (Fig. 2d) resembles identical features with the pristine glass, suggesting that the vitrification of the PS layer on the upper surface leaves the phosphate glass network unaffected. The corresponding cross-sectional spectrum within the vitrified PS layer exhibits the four characteristic peaks of PS at 953, 998, 1366, and 1593 cm^−1^, along with those of the phosphate glass at 675 and 1144 cm^−1^.^18,22^ This implies that the vitrified PS layer co-exists within the phosphate glass network. Nevertheless, a closer inspection of the Raman spectrum within the encapsulated PS layer region reveals an increase in the relative intensity of the 675 cm^−1^ peak relative to the 1144 cm^−1^ peak, suggesting a reduction of the terminal oxygen entities within the phosphate backbone relative to the bridging entities. This is rationalized if one considers the binding of some of the negatively charged terminal oxygen atoms of the glass network to the PS aromatic rings by means of typical nucleophilic aromatic substitution.^23^ Notably, such a process becomes more intense moving towards the surface, as it is revealed from the top-view Raman profile of the PS layer glass (Fig. 3b). The latter exhibits the glass network bridging oxygen peak at 675 cm^−1^, whereas the terminal oxygen atom peak is almost absent, suggesting the intensive binding of the negatively charged oxygen atoms to the aromatic rings of the encapsulated PS.

Along similar lines, Fig. 3c depicts the captured Raman profiles of the so-formed PS powder composite glass along with those of the pristine glass. As expected, the spectra within the pristine AgPO_3_ glass region mainly exhibit the two typical vibration modes of the phosphate entities as described above. Notably, the cross-sectional Raman spectra within the embedded PS core are dominated by the PS-originating modes at 953, 998, 1366, and 1593 cm^−1^, while the phosphate entity peaks are almost absent. In contrast to what was the case for the PS layer glasses, this observation for the PS powder platforms is attributed to the high density of the PS powder within the embedded PS core (Fig. 2f), when compared to the more effective mixing of the much thinner drop-cast PS film of the PS layer samples (Fig. 2d).

Although the incorporation of PS in the glass does not lead to any significant structural changes in the embedded PS and the phosphate network, it has a clear impact on the optical properties of the composite glasses. Fig. 3d presents the optical absorbance spectra of PS/MeOH solutions at ambient temperature and after heating at 80 °C, along with a typical profile of a PS layer composite glass. Consistent with earlier studies, the pristine binary AgPO_3_ glass is nearly transparent down to a wavelength of 380 nm. In contrast, PS exhibits three distinct absorption peaks at 278 nm, 355 nm, and 407 nm. The lowest-energy absorption band is attributed to transitions involving the orientation of the transition dipole moment along the long axis passing through the 2- and 6-phenyl groups. In contrast, the higher-energy bands are associated with transitions involving the dipole moment orientation along the 4-phenyl group of the salt.^18^ Notably, heating beyond the methanol evaporation point appears to leave these absorption spectra unaffected (Fig. 3d). However, when the PS is encapsulated within the glass matrix, the absorption profile exhibits a noticeable red shift. This can be attributed to several physical phenomena. Silver ions (Ag^+^) from the AgPO_3_ matrix can form weak coordination interactions with the oxygen atoms of the pyrylium ring. This perturbs the electronic states, leading to a red shift in absorption.^24^ Additionally, the pyrylium ion has a delocalized positive charge, which can interact with the negatively charged phosphate groups (PO_3_^−^) in the glass matrix. This modifies the electron density distribution, affecting the π → π* transitions and shifting the absorption to longer wavelengths.^25^

The fabricated composite PS-glass architectures demonstrate remarkable optical waveguide properties, highlighting the potential of the developed synthesis approach for advanced optoelectronic device fabrication. Specifically, waveguide devices were prepared from both solution- (PS layer-glass) and powder- (PS powder-glass) based samples by forming defined waveguide paths within the glass matrix. This process enabled precise, spatially controlled modification of the refractive index of the composite glass. The fabrication method, illustrated in Fig. S6, involves attaching a thin tape onto the glass surface to define the waveguide channels. The upper section of Fig. S6 shows the preparation of waveguides *via* the PS/MeOH solution deposition, solvent evaporation and PME, while the lower section displays the powder-based method, wherein solid PS was spattered at the tape–sample interface and then encapsulated within the glass through PME.

**Fig. 4:**
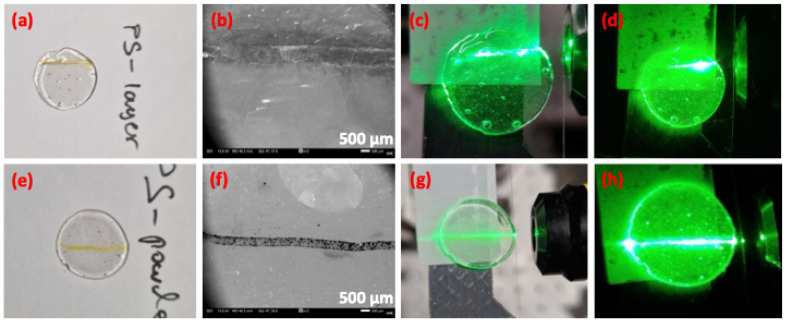
(a) Photo of the PS layer composite glass. (b) SEM image of the PS layer composite glass. (c) Green light waveguiding through the PS layer glass with lights on. (d) Green light waveguiding through the PS layer glass with lights off. (e) Photo of the PS powder composite glass. (f) SEM image of the PS powder composite glass. (g) Green light waveguiding through the PS powder glass with lights on. (h) Green light waveguiding through the PS powder glass with lights off.

Fig. 4a presents a photo of the PS layer-glass waveguide, while Fig. 4b depicts the corresponding SEM image, clearly illustrating the formation of the PS pathway embedded in the glass. Based on the tape-defined pattern (Fig. S6a), the waveguide channel has a width of around 700 μm, with a depth of around 50 μm (Fig. S7c). Similar PS pathway dimensions were obtained for the PS-powder glass (Fig. S7d–f). Such PS thicknesses in both devices were the minimum that could be obtained upon employing the described tape method for the formation of the waveguide pathways. Noticeably, the thickness increases when compared to samples without the tape method (Fig. 2), since the employed tape increases the sideways pressure resulting in deeper penetration lengths of the PS inside the host glass.

Fig. 4c demonstrates green light transmission through the PS layer-glass waveguide, achieved by incorporating a CW laser emitting at 526 nm into the PS pathway *via* a microscope objective (Fig. S8). Likewise, Fig. 4e presents a PS powder-glass waveguide, with Fig. 4f depicting its SEM profile. The robust waveguiding capability of the PS powder-based glass is clearly demonstrated in Fig. 4g and h, taken with laboratory lights on and off, respectively. To further illustrate the waveguiding performance, a video (Video S1) of the waveguiding process was captured. Initially, the laser beam is positioned outside the vitrified PS waveguide channel, resulting in no guided light. However, at around 8 seconds into the video, the laser beam is precisely repositioned into the high-index PS pathway using an *x*,*y*,*z* translation stage (Fig. S8), resulting in intense waveguided green light propagating through the composite glass (Video S1 and Fig. 4c, d). The video further demonstrates waveguide efficiency by repeatedly moving the laser beam in and out of alignment with the waveguide channel entrance. Furthermore, Fig. S9a demonstrates the waveguiding performance when the PS-glass device is intentionally tilted by approximately 4° relative to the horizontal laser beam direction. Under this tilted configuration, the coupled green light follows the angled PS pathway, clearly deviating from the initial horizontal laser trajectory.

The obtained waveguiding features rely on the principle of total internal reflection (TIR).^26^ TIR occurs when light propagating from a medium with a higher refractive index (denser medium) encounters an interface with a lower refractive index (rarer medium) at an angle exceeding the critical angle, causing complete reflection within the denser medium. In the developed devices, the encapsulated PS regions serve as the optically denser waveguiding cores, while the surrounding host AgPO_3_ glass (with a refractive index of approximately 1.7)^26^ serves as the rarer cladding medium. This refractive index contrast directly influences the optical losses observed in the two fabricated waveguide devices (Fig. S9b). Namely, the PS layer-glass waveguide exhibits higher losses (∼6 dB cm^−1^), around four times greater than those measured in the PS powder-based device (∼1.5 dB cm^−1^). This difference is consistent with structural and compositional analyses provided by SEM (Fig. 2) and Raman spectroscopy (Fig. 3), which confirm that the PS powder-based waveguide contains a much denser concentration of the vitrified PS salt compared to the PS layer-based device formed from the diluted PS/MeOH solution. However, it is highlighted that the obtained thickness fluctuation of the encapsulated PS pathway between the two types of devices also affects the obtained losses and must be taken into account when comparing the waveguide efficiency of the developed devices.

The waveguiding properties observed in the developed PS-glass platforms can be explained by considering both the intrinsic optical characteristics of individual pyrylium molecules and the collective effects resulting from their dense molecular packing. In isolation or dilute solvent environments, individual pyrylium molecules typically exhibit refractive indices of around 1.45, comparable to values reported for structurally related aromatic salts such as pyridinium derivatives.^27,28^ These values arise from their conjugated aromatic structure, known for significant electronic polarizability due to π-electron delocalization. However, when pyrylium molecules are densely confined within the phosphate glass matrix, the collective effects of intermolecular interactions and increased packing density lead to a considerable enhancement of the effective refractive index. This phenomenon is analogous to observations in conjugated polymer systems, where high refractive indices, often ranging between 1.75 and 2, result from the combined effects of ordered aromatic units, strong intermolecular interactions, and dense molecular packing.^29,30^ Although polymers and small molecules differ structurally, the underlying principle remains consistent: increased packing density enhances electronic polarizability and reduces free volume, resulting in elevated refractive indices. In the developed PS-glass powder-based devices, the refractive index of the embedded PS region at 526 nm was calculated to be 1.92. The resulting elevated refractive index contrast between the densely packed pyrylium salt pathways (core) and the lower-index phosphate glass (cladding, with a refractive index of ∼1.7) effectively creates a robust waveguiding structure. This explains why the waveguiding performance is notably superior in PS powder-based devices, where the considerably denser molecular packing of PS forms a more effective optical pathway compared to that in solution-derived PS-layer devices.

## 4. Conclusions

In summary, we demonstrated a novel materials-integration strategy by successfully incorporating, for the first time, a functional pyrylium salt directly into an inorganic phosphate glass matrix, enabling the fabrication of optical waveguide architectures unattainable by conventional techniques. The developed feasible, low-temperature, post-melting synthesis method allows spatially controlled embedding of the pyrylium salt, resulting in clearly defined high-refractive-index pathways capable of efficiently guiding light by total internal reflection. Importantly, the pyrylium salt preserves its intrinsic optical properties within the stable glass host, indicating the robustness and scalability of the PME process. Unlike traditional waveguide fabrication techniques, such as laser inscription, chemical etching, or lithography, the proposed approach avoids the need for expensive and complex equipment, making it a highly cost-effective method for producing integrated photonic devices. The resulting composite glass waveguides are fully solid-state, inherently durable, and thus particularly suitable for practical optoelectronic applications where conventional polymer-embedded organic dyes typically degrade or fail over time. This work establishes a versatile platform technology that could readily be extended to a diverse range of organic or organometallic salts and dyes, covering a broad spectrum of optical wavelengths and functionalities, including enhanced luminescence, nonlinear optics, or stimulated emission properties. It thus opens avenues for a new class of hybrid organic–inorganic photonic devices, harnessing the extensive library of organic functional molecules within robust glass architectures, unlocking a diverse range of applications for next-generation photonic circuits and optoelectronic systems.

## Conflicts of interest

There are no conficts to declare.

## Supplementary Material

NR-017-D5NR02213D-s001

NR-017-D5NR02213D-s002

## Data Availability

The data supporting the findings of this study are available within the article and its SI. Additional data related to this research are available upon reasonable request from the corresponding author. SI consists of Figures and Videos. See DOI: https://doi.org/10.1039/d5nr02213d
