# Supplementary material for: Advanced optical waveguide design via encapsulation of 2,4,6-triphenylpyrylium chloride in oxide glasses
Source: Nanoscale. 2025 Jul 31;17(35):20148–56. doi: 10.1039/d5nr02213d (PMC12355649; doi:10.1039/d5nr02213d)
Supplement: NR-017-D5NR02213D-s001 [file NR-017-D5NR02213D-s001.pdf]

Supplementary Information (SI) for Nanoscale.
This journal is © The Royal Society of Chemistry 2025

Supporting Information

Advanced Optical Waveguide Design via Encapsulation of 2,4,6-Triphenylpyrylium Chloride in Oxide Glasses

Eleni Agapaki[1], Ioannis Konidakis*[1], Egor Evlyukhin[1], Klytaimnistra Katsara[1], Georgios Kenanakis[1], David King[2], Haesook Han[2], Pradip K. Bhowmik[2] and Emmanuel Stratakis*[1]

[1]. Institute of Electronic Structure and Laser (IESL), Foundation for Research and Technology-Hellas (FORTH), 70013 Heraklion-Crete, Greece.

[2]. Department of Chemistry and Biochemistry, University of Nevada Las Vegas, Las Vegas, Nevada 89154, United States.

***Corresponding authors:** ikonid@iesl.forth.gr, stratak@iesl.forth.gr

Figures

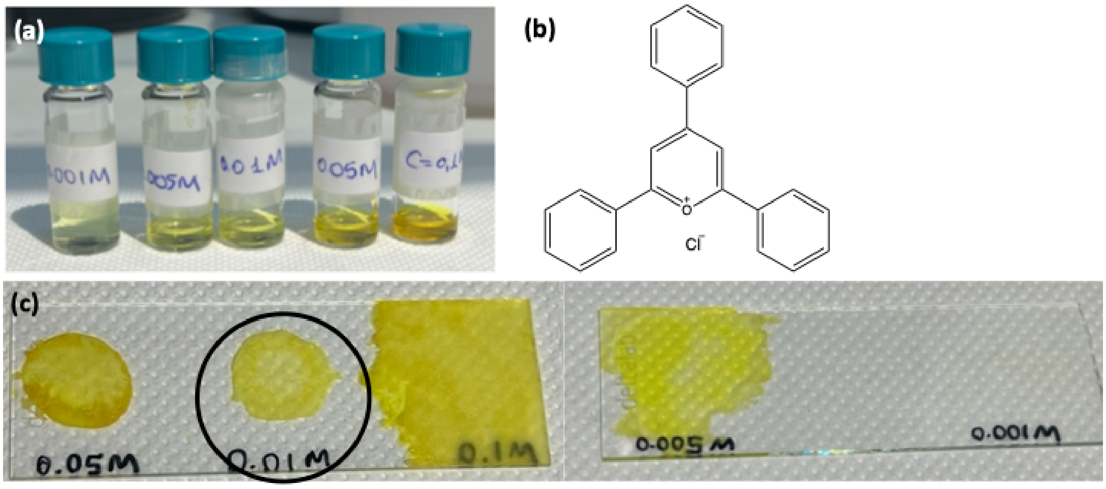

Fig. S1: **(a)** Solutions of the 2,4,6-triphenylpyrylium chloride salt diluted in MeOH in several concentrations. **(b)** 2,4,6-triphenylpyrylium chloride salt chemical structure. **(c)** 2,4,6-triphenylpyrylium chloride salt in several concentrations, drop-casted on microscopic glass following solvent evaporation.

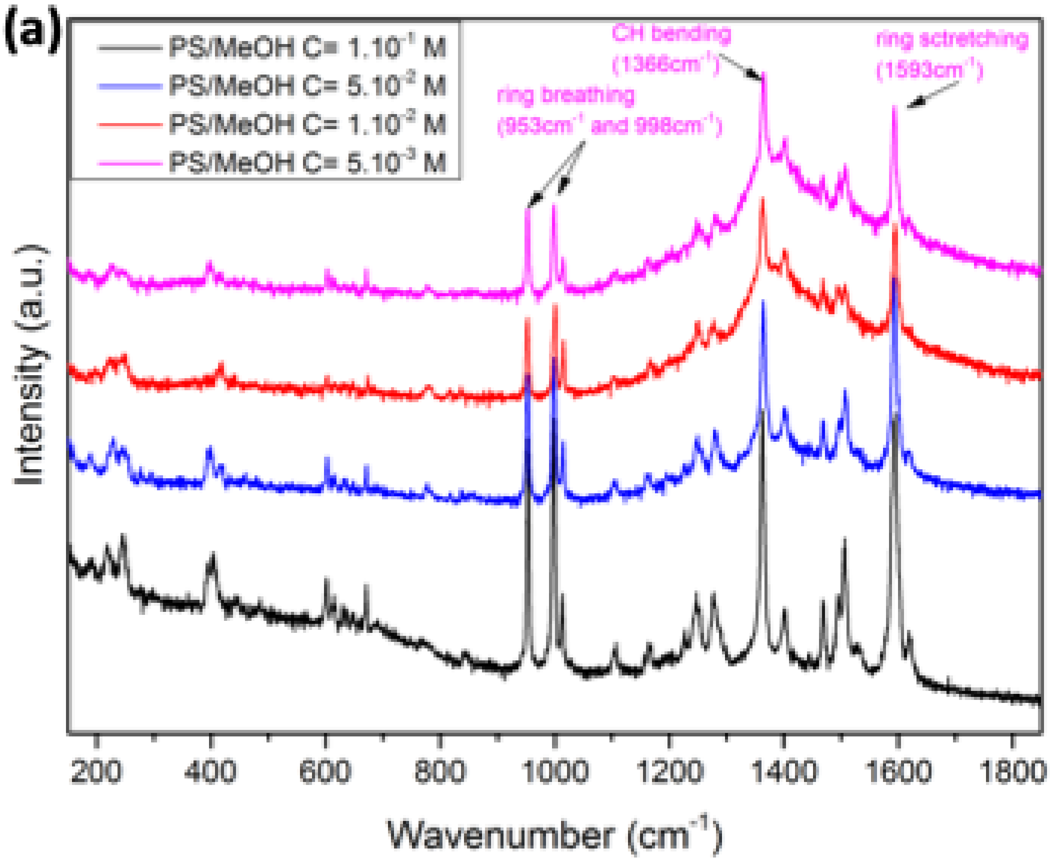

Fig. S2: **(a)** Raman spectra of drop-casted 2,4,6-triphenylpyrylium salt (PS) layers on typical microscope slide glasses upon MeOH evaporation prepared from various concentration of PS/MeOH solutions.

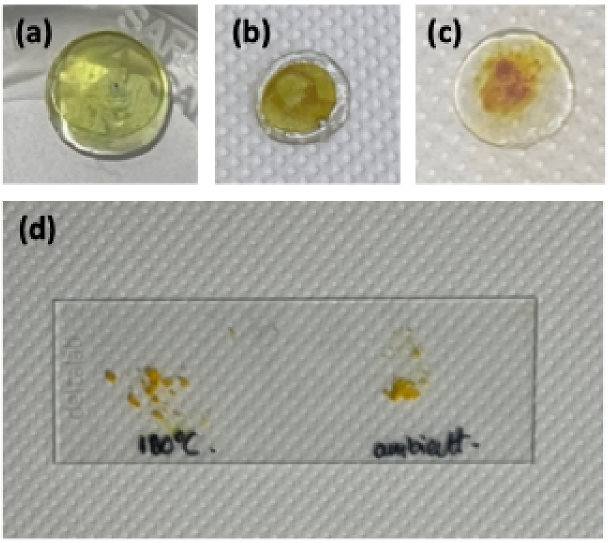

Fig. S3: Photos of 2,4,6-triphenylpyrylium chloride salt (PS) diluted in MeOH ($C=10^{-3}M$) and encapsulated in glass **(a)**. Diluted in MeOH ($C=10^{-2}M$) and encapsulated in glass **(b)**. **(c)** Powder (3 mg) encapsulated in glass. **(d)** PS powder at ambient temperature (right) and at 180°C (left).

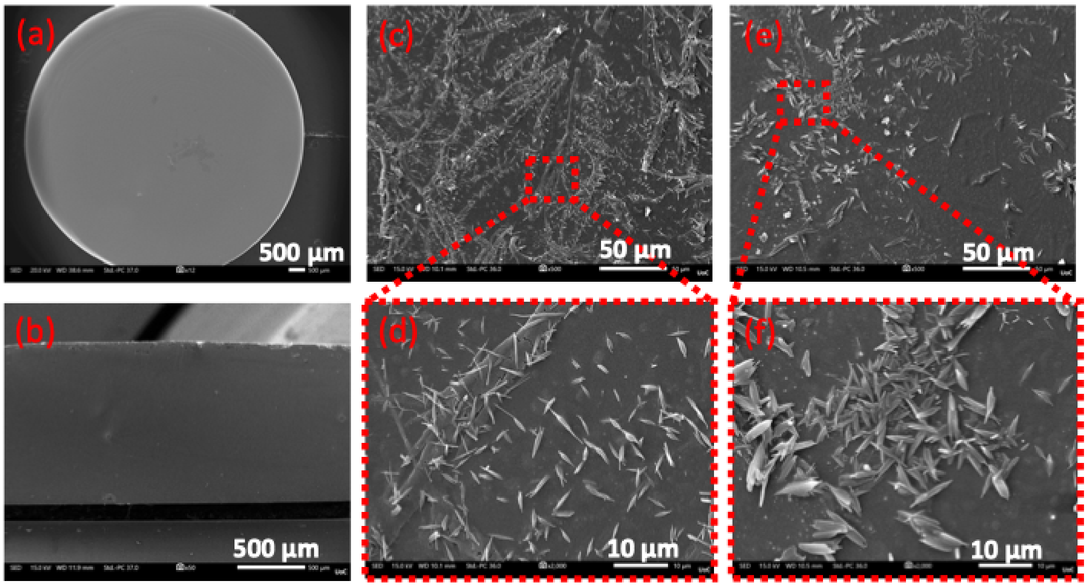

Fig. S4: SEM images showing: **(a)** a top view of pristine $AgPO_3$ glass. **(b)** a cross section of pristine $AgPO_3$ glass. **(c)** 2,4,6-triphenylpyrylium chloride salt diluted in MeOH ($C = 10^{-2}$ M) at ambient temperature. **(d)** a magnified image of **(c)**. **(e)** 2,4,6-triphenylpyrylium chloride salt diluted in MeOH ($C = 10^{-2}$ M) at 80°C. **(f)** a magnified image of **(e)**.

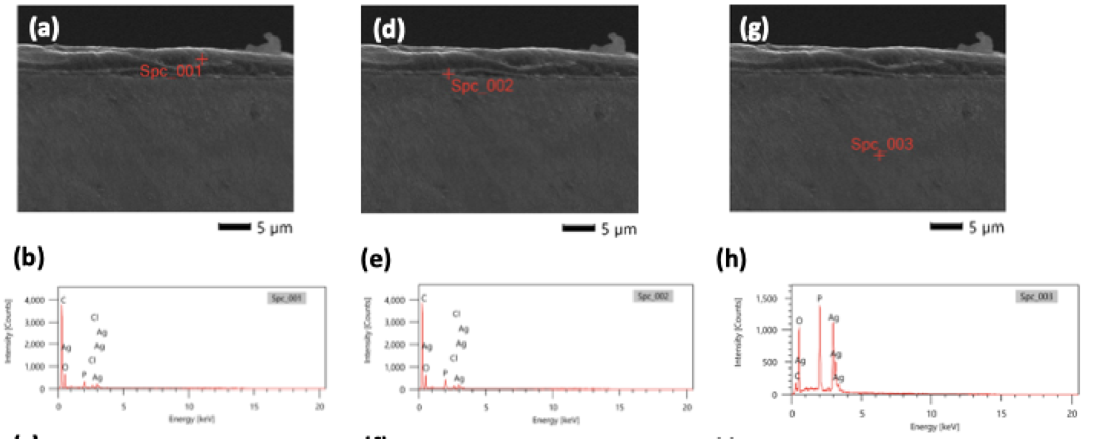

(c)

Element	Line	Mass%	Atom%
C	K	68.07±0.41	77.88±0.47
O	K	23.20±0.58	19.93±0.50
P	K	2.41±0.09	1.07±0.04
Cl	K	1.23±0.07	0.48±0.03
Ag	L	5.09±0.21	0.65±0.03
Total		100.00	100.00
Spc_001			Fitting ratio 0.0184

(f)

Element	Line	Mass%	Atom%
C	K	69.31±0.41	78.90±0.46
O	K	21.96±0.56	18.76±0.48
P	K	3.11±0.10	1.37±0.05
Cl	K	0.98±0.06	0.38±0.02
Ag	L	4.65±0.20	0.59±0.02
Total		100.00	100.00
Spc_002			Fitting ratio 0.0188

(i)

Element	Line	Mass%	Atom%
C	K	5.20±0.17	11.95±0.39
O	K	37.07±0.73	63.92±1.27
P	K	14.76±0.27	13.15±0.24
Ag	L	42.97±0.67	10.99±0.17
Total		100.00	100.00
Spc_003			Fitting ratio 0.0303

Fig. S5: SEM and corresponding EDS data of 2,4,6-triphenylpyrylium chloride layer encapsulated in $AgPO_3$ glass in **(a)**, **(b)**, **(c)**. On the PS layer glass interface **(d)**, **(e)**, **(f)**. On the silver phosphate glass matrix region of the PS-glass **(g)**, **(h)**, **(i)**.

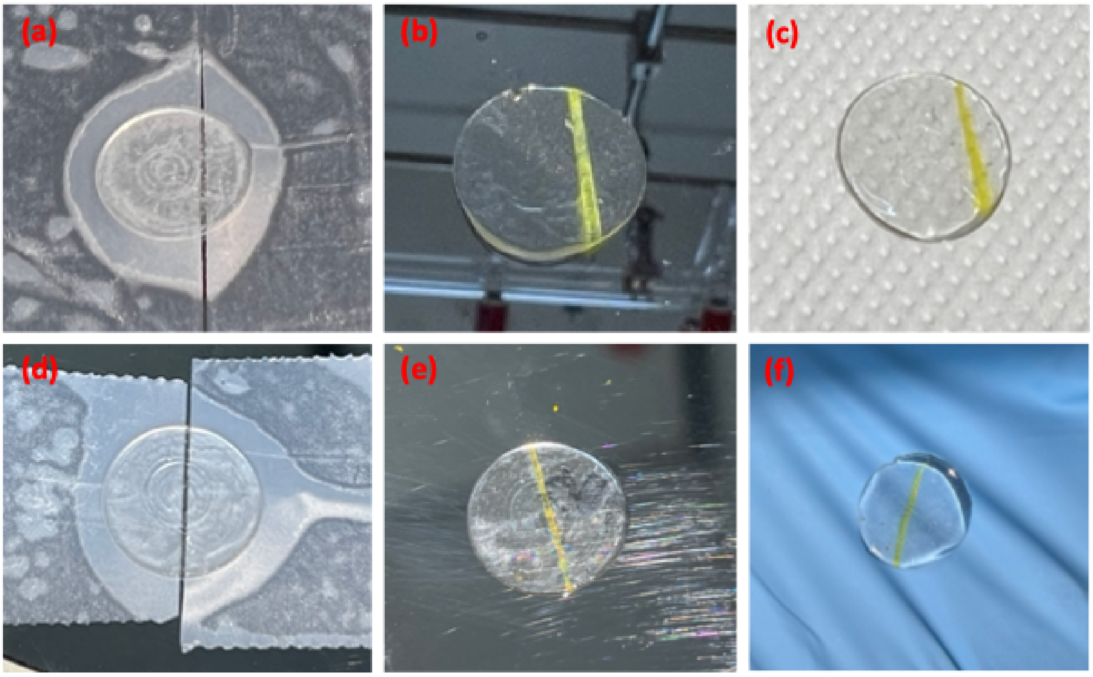

Fig. S6: Illustration of the preparation of the waveguide devices. **(a)** Tape mounted for PS layer pathway development. **(b)** Deposition of the PS layer from the PS/MeOH solution. **(c)** PS layer glass waveguide device after encapsulation. **(d)** Tape mounted for the PS powder pathway development. **(e)** Deposition of the PS powder on the glass surface. **(f)** PS powder glass waveguide device after encapsulation.

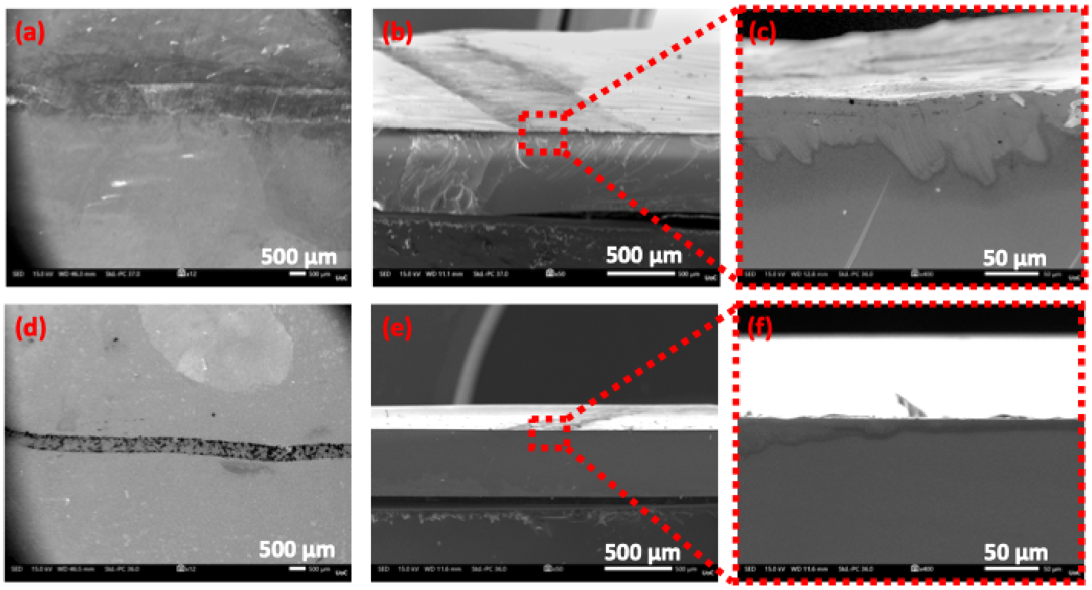

Fig. S7: SEM images of **(a)** 2,4,6-triphenylpyrylium chloride salt (PS) layer waveguide device, top view. **(b)** PS layer waveguide device, cross section. **(c)** Magnified image of **(b)**. **(d)** PS salt powder waveguide device, top view. **(e)** PS powder waveguide device, cross section. **(f)** Magnified image of **(e)**.

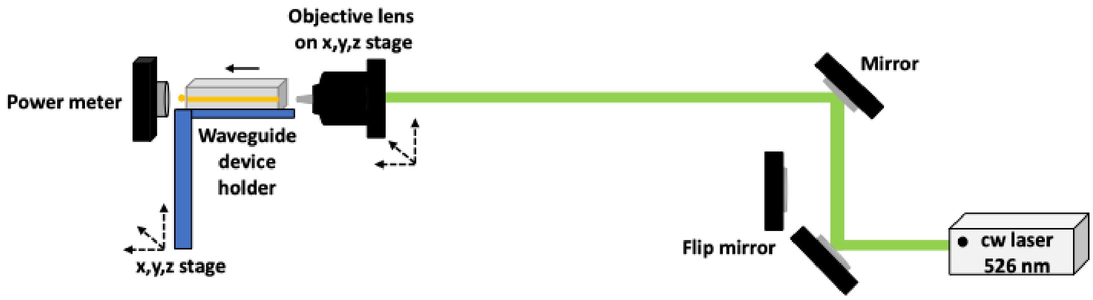

Fig. S8: Depiction of experimental setup with the necessary optical components for guiding the cw laser beam towards the waveguide device.

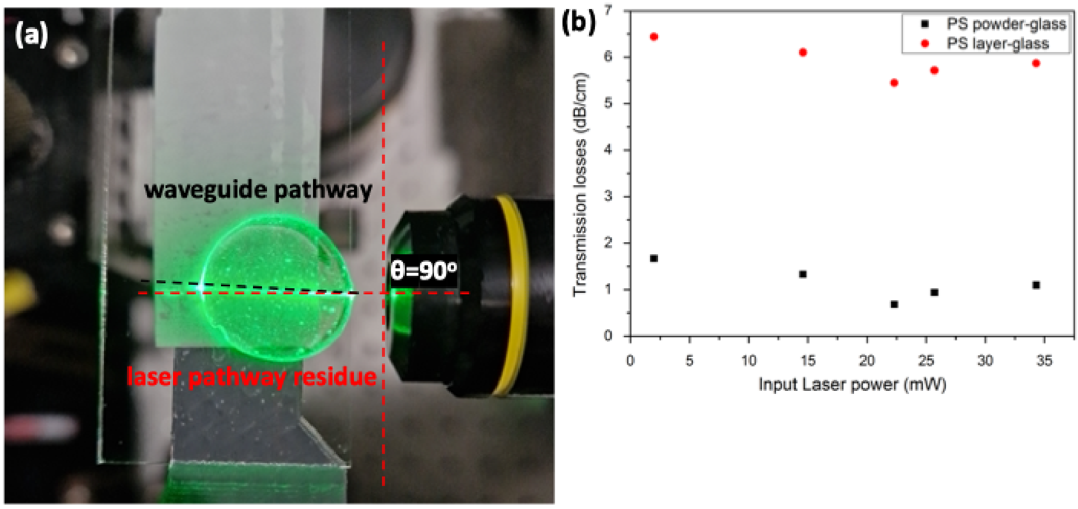

Fig. S9: (a) Green laser beam focused outside the waveguide pathway, and thus, forming a 90° angle with respect to the objective. **(b)** Measured optical losses of both waveguides, i.e., PS layer glass and PS powder glass devices.

Videos:

Vid. S1: Demonstration of the PS-powder composite glass waveguide features upon coupling in and out of the cw 526 nm laser beam.
